# Evaluation of prenatal diagnosis of congenital anomalies diagnosable by prenatal ultrasound in patients in neonatal intensive care units of Cali, Colombia

**Published:** 2014-03-30

**Authors:** Wilmar Saldarriaga-Gil, Fabián Andrés Ruiz-Murcia, Andrés Fandiño-Losada, Manuel Enrique Cruz-Perea, Carolina Isaza de Lourido

**Affiliations:** 1 Department of Morphology Faculty of Health, Universidad del Valle. Cali, Valle, Colombia. carolinaisadel@gmail.com; 2 Department of Gynecology & Obstetrics Faculty of Health, Universidad del Valle. Cali, Valle, Colombia; 3 Group on Congenital Malformations and Dysmorphology, Faculty of Health. Universidad del Valle (MACOS). Cali, Valle Colombia.; 4 School of Public Health, Universidad del Valle. Cali, Valle, Colombia. carlos.fandino@correounivalle.edu.co

**Keywords:** Congenital anomalies, prenatal diagnosis, prenatal ultrasound, accessibility to health services

## Abstract

**Introduction:**

The study aim was to determine the frequency of prenatal ultrasound diagnosis of congenital anomalies in Newborns (NB) with birth defects hospitalized in two Neonatal Intensive Care Units (NICU) of Cali (Colombia) and to identify socio-demographic factors associated with lack of such diagnosis.

**Patients and methods:**

It was an observational cross-sectional study. NB with congenital defects diagnosable by prenatal ultrasound (CDDPU), who were hospitalized in two neonatal intensive care units (NICU), were included in this study. A format of data collection for mothers, about prenatal ultra-sonographies, socio-demographic data and information on prenatal and definitive diagnosis of their conditions was applied. Multiple logistic and Cox regressions analyses were done.

**Results:**

173 NB were included, 42.8% of cases had no prenatal diagnosis of CDDPU; among them, 59.5% had no prenatal ultrasound (PNUS). Lack of PNUS was associated with maternal age, 25 to 34 years (Odds Ratio [OR]: 4.41) and 35 to 47 years (OR: 5.24), with low levels of maternal education (OR: 8.70) and with only a PNUS compared to having two or more PNUS (OR: 4.00). Mothers without health insurance tend to be delayed twice the time to access the first PNUS in comparison to mothers with payment health insurance (Hazard Ratio [HR]: 0.51). Among mothers who had PNUS, screening sensitivity of CDDPU after the 19^th^ gestational week was 79.2%.

**Conclusions:**

The frequency of prenatal diagnosis is low and is explained by lack of PNUS, or by lack of diagnostic in the PNUS. An association between lack of PNUS and late age pregnancy and low level of maternal education was found. In addition, uninsured mothers tend to delay twice in accessing to the first PNUS in comparison to mothers with health insurance. It is necessary to establish national policies which ensure access to appropriate, timely and good quality prenatal care for all pregnant women in Colombia.

## Abstract (es)

**Introducción:**

El objetivo del estudio fue determinar la frecuencia de diagnóstico ecográfico prenatal de anomalías congénitas en Recién Nacidos (RN) con defectos congénitos hospitalizados en dos Unidades de Cuidado Intensivo Neonatal (UCIN) de Cali (Colombia) e identificar factores sociodemográficos relacionados con la ausencia del mismo.

**Pacientes y métodos:**

Estudio observacional tipo transversal. Se incluyeron RN con Defectos Congénitos Diagnosticables por Ecografía Prenatal (DCDEP) hospitalizados en unidad de cuidados intensivos neonatal. Se aplicó un formato de recolección de datos a las madres, sobre realización de ecografías, datos sociodemográficos e información sobre el diagnóstico prenatal y definitivo de su patología. Se realizaron análisis de regresión logística y de regresión de Cox múltiples.

**Resultados:**

Se incluyeron 173 recién nacidos, el 42.8% de los casos no tenía diagnóstico prenatal, de éstos, el 59.5% no tenía ninguna Ecografía Prenatal (ECOPN). La ausencia de ECOPN se asoció con la edad materna de 25 a 34 años (Oportunidad Relativa [OR]: 4.41) y de 35 a 47 años (OR: 5.24), con bajo nivel de escolaridad materna (OR: 8.70) y con sólo una ECOPN en comparación con tener dos o más (OR: 4.00). Las madres no aseguradas tienden a demorarse el doble del tiempo en acceder a la primera ECOPN en comparación con gestantes del régimen contributivo (Peligro Relativo [HR]: 0.51). Entre las madres que se realizaron ECOPN, la sensibilidad del tamizaje de DCDEP después de la semana 19 de gestación fue 79.2%.

**Conclusiones:**

La frecuencia de diagnóstico prenatal es baja y se explica por la ausencia de ECOPN o por la falta de diagnóstico en la ecografía. Se encontró una asociación entre la no realización de ECOPN y gestantes tardías y de bajo nivel de escolaridad. Además, las madres no aseguradas tienden a demorarse el doble del tiempo en acceder a la ECOPN en comparación con las madres aseguradas. Se deben establecer políticas nacionales que garanticen el acceso a un apropiado control prenatal y ecografías oportunas y de buena calidad para todas las embarazadas en Colombia.

## Introduction

The routine use of *prenatal ultrasound* (**PNUS**) has been standardized with the object of making an approach to securing the health of the embryo or fetus, establishing gestational age, establishing if the embryo or fetus is alive, detecting congenital anomalies, while identifying multiple pregnancies, fetal growth disorders and placental disturbances 1 ^,^ 2 ^,^ 3. With the development of equipment for ultrasound and magnetic resonance imaging along with the software to manipulate images, the training of personnel to gather and interpret images, such as specialists in perinatology and maternal medicine, virtually 100% of anatomical birth defects could be diagnosed before birth 3. With a prenatal diagnosis of anomalies the prognosis improves for affected newborns, and it allows for *in utero* interventions and preparation of neonatology team in advance for caring the newborn and preparing the family regarding its new member with special needs 1 ^,^ 3 ^,^ 4. Additionally, it allows for the voluntary option of abortion in cases incompatible with life in those countries where it is regulated 3.

However, the frequency of prenatal diagnoses in low-risk populations and the fulfillment of screening protocols are far from ideal. The Radius study in 1993 reported a correct prenatal diagnosis of major abnormalities in 34.8% of cases in the group undergoing screening with ultrasound and 11% in the control group 1. In Latin America, Capaña *et al.,* found a prenatal diagnosis in 56% of cases from 18 hospitals in 4 countries 4. In Colombia, Gomez *et al*., reported the presence of prenatal diagnoses in 32% of newborns with congenital birth defects diagnosed before discharge 5.

In our settings, newborns with birth defects hospitalized in *neonatal intensive care units* (**NICU**) are often seen without a prenatal diagnosis even though a diagnosis may be performed via obstetric ultrasound. This implies that the care of the newborn is not previously planned and, therefore, a subsequent reduction in associated morbidity and mortality is not possible to accomplish. 

The objective of the study was to determine the frequency of prenatal ultrasound diagnoses of subsequent newborns with congenital birth defects who were hospitalized on two neonatal intensive care units in Cali. It is also to identify the socio-demographic factors related to its absence (i.e. lack of access). Thus, the intent is to quantify the number of patients lacking prenatal diagnosis of birth defects diagnosable by ultrasound, along with identifying factors that might be leveraged to improve accessibility and the quality of prenatal diagnoses.

## Matherials and Methods 

An observational cross-sectional study was conducted. The study population included inpatients in neonatal intensive care units in two tertiary-level institutions in the city of Cali, Colombia, between November 1, 2010 and February 29, 2012. The first was a reference hospital in the public network for Southwestern Colombia and the second was a private hospital. Among all hospitalized patients in such NICU, subjects with *congenital birth defects diagnosable by prenatal ultrasound* (**CDDPU**) were included in this study. Thus, a list of CDDPU anomalies for study inclusion was compiled. Case data were initially taken from the clinical records, along with any postnatal diagnoses of birth defects; a format for data collection from mothers was applied which included information on the number and outcomes of prenatal ultrasounds along with several relevant socio-demographic variables.

Data were tabulated on Epidata^®^ and were analyzed with Stata 11^®^. The frequencies of the results were determined according to the objectives. Measures of association (i.e. Odds Ratio: OR) were calculated using multiple logistical regressions. Additionally, the Hazard Ratio (HR) was determined for timely access to a PNUS with respect to different explanatory variables by means of survival analysis with multiple Cox regression.

The sensitivity of ultrasound screening was established from the number of cases diagnosed with CDDPU divided by the number of mothers who underwent PNUS after the 19^th^ week of gestation. It should be noted that mothers with only one ultrasound which were included in the analysis, had it performed after the 19^th^ week of gestation. This project was approved by the Ethics Committees of Universidad del Valle (Colombia) and the two hospitals.

## Results

A total of 173 cases were included. Of these, 57.2% had a positive PNUS with at least one CDDPU. The total number of CDDPU in the study was 217, with an average of 2.2 CDDPU per patient (see Table 1, for the description of congenital defects). The 42.8% (95% Confidence Interval [CI]: 35.3-50.5) of patients had not prenatal diagnosis of CDDPU. Among these 74 patients, the 59.5% (95% CI: 47.4-70.7) had not prenatal ultrasound (PNUS), and the 2.3% (4 patients) had only a single ultrasound performed before the 19^th^ gestational week. The remaining 125 patients had at least one PNUS at the 19^th^ week of gestation or later, but among them 20.8% (95% CI 14.1 - 29.0) had no CDDPU diagnosis despite such ultrasounds.

**Table 1.**
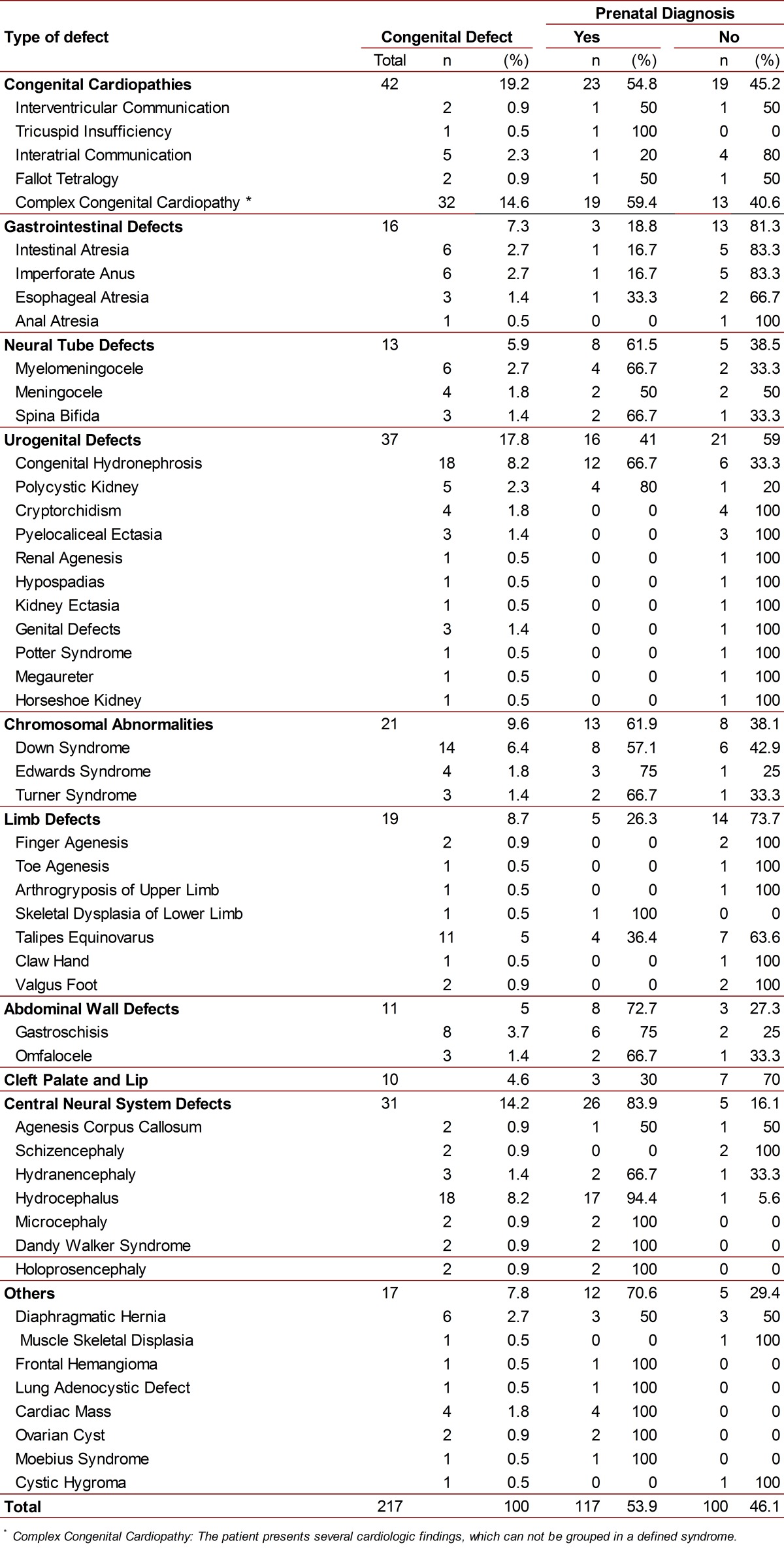
Frequencies of congenital birth defects found and frequency of their prenatal diagnoses by ultrasound

Therefore, the sensitivity of the screening process (including at least one PNUS at the 19^th^ week of gestation or later) was 79.2% (95% CI: 71.0 - 85.9). There were no statistically significant differences in the sensitivity of screening by PNUS according to the type of expectant women's health insurance.

Multiple logistic regression analyses indicated that lack of PNUS was associated with maternal age in the groups 25-34 and 35-47 years old when compared with the group of 19-24 years old mothers, and also it was associated with the educational level of pregnant women, i.e. incomplete elementary school or less (see Table 2).

**Table 2.**
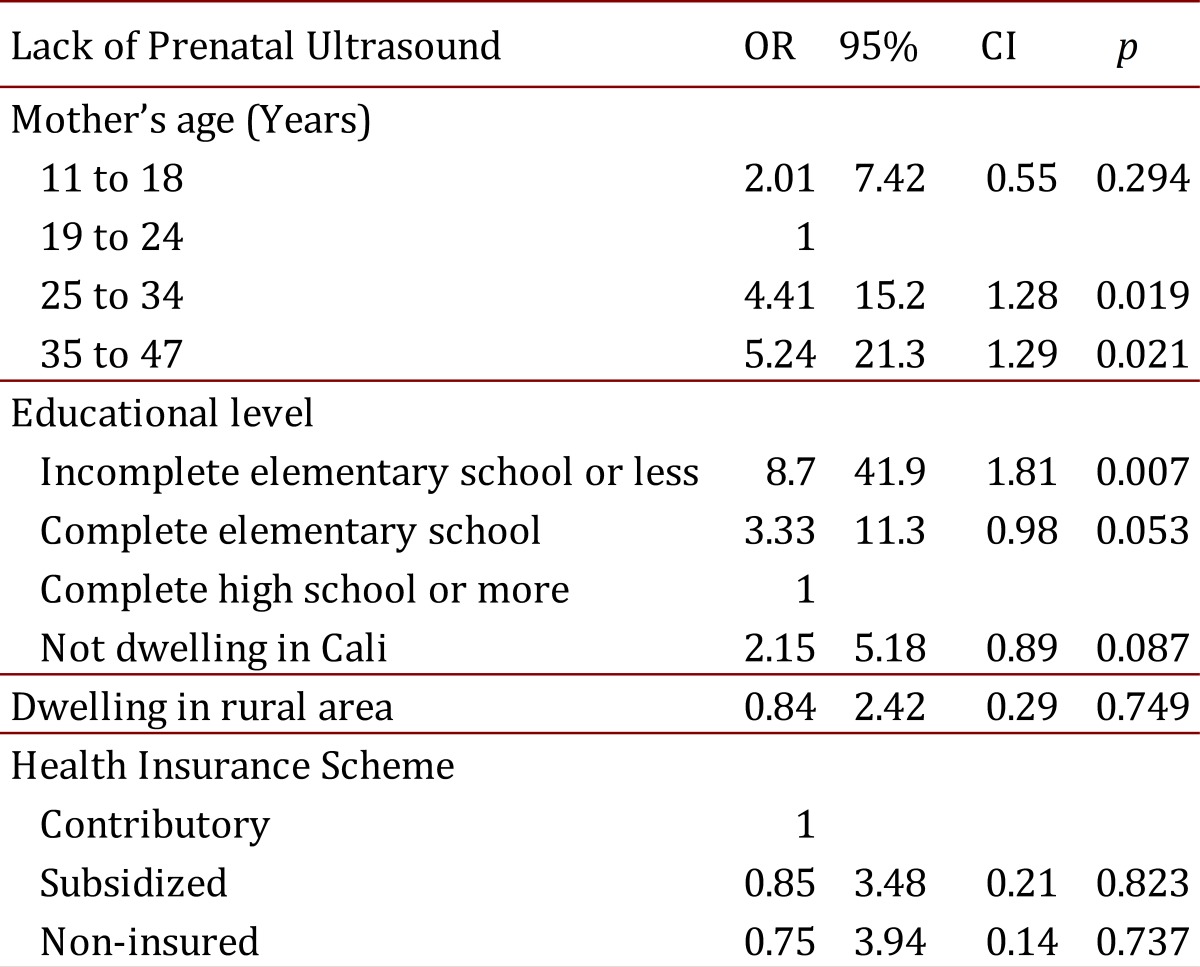
Factors Associated with Lack of Prenatal Ultrasound during the Pregnancy

Among the mothers who underwent at least one PNUS at the 19^th ^gestational week or later, it was found that the group of mothers of 25 to 34 years old had a greater odds of having a diagnosis of CDDPU when compared to the 19 to 24 years old group (OR: 3.70, 95% CI: 1.04-12.50), although such association was not significant after controlling for educational level, welling area (rural and/or outside Cali) and type of health insurance. It should be noted that among all (100%) mothers of 35 years or older who accessed to at least one PNUS (whose offspring indeed had a congenital defect), at least one CDDPU was detected in the PNUS screening process of each patient.

On the other hand, it was found that having only one PNUS in comparison with having two or more increased fourfold the risk of not having a prenatal diagnosis (OR: 4.00, 95% CI :1.02-15.77).

**Figure 1.**
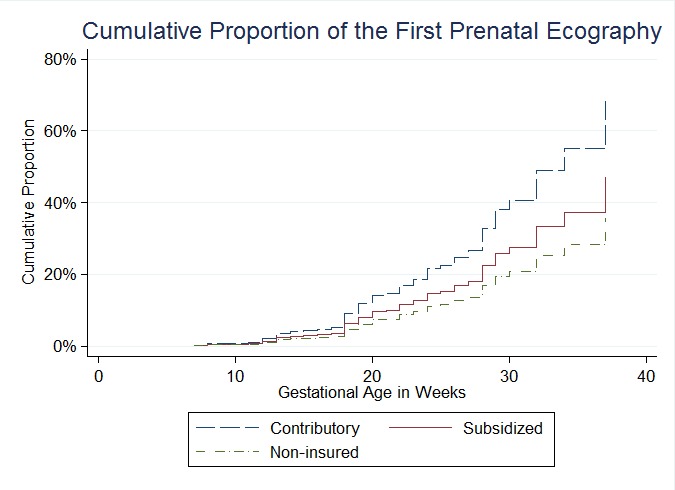
Cumulative Proportion of Access to the First Prenatal Ultrasound during the Pregnancy Control over the Gestational Age, by Health Insurance Scheme.

We investigated the factors associated with having only one PNUS, and a marginally significant relationship was observed for mothers belonging to the subsidized health insurance compared to mothers with health insurance by payment, i.e. the contributory scheme, according to the Colombian health system jargon (OR: 4.76, *p*= 0.058). A similar trend was observed among pregnant women who were uninsured, but it was not statistically significant. Additionally, uninsured mothers tended to delay the access to the first PNUS twice as long as mothers of the contributory scheme did (HR: 0.51, 95% CI: 0.27-0.98). This trend was also observed among mothers belonging to the subsidized insurance, although it was not statistically significant (see Fig. 1). The adjusted analysis indicated that such relationship was explained by the lower educational levels of pregnant women (see Table 3). The 75% of mothers without health insurance, in this study, had an educational level of elementary school or less.

**Table 3.**
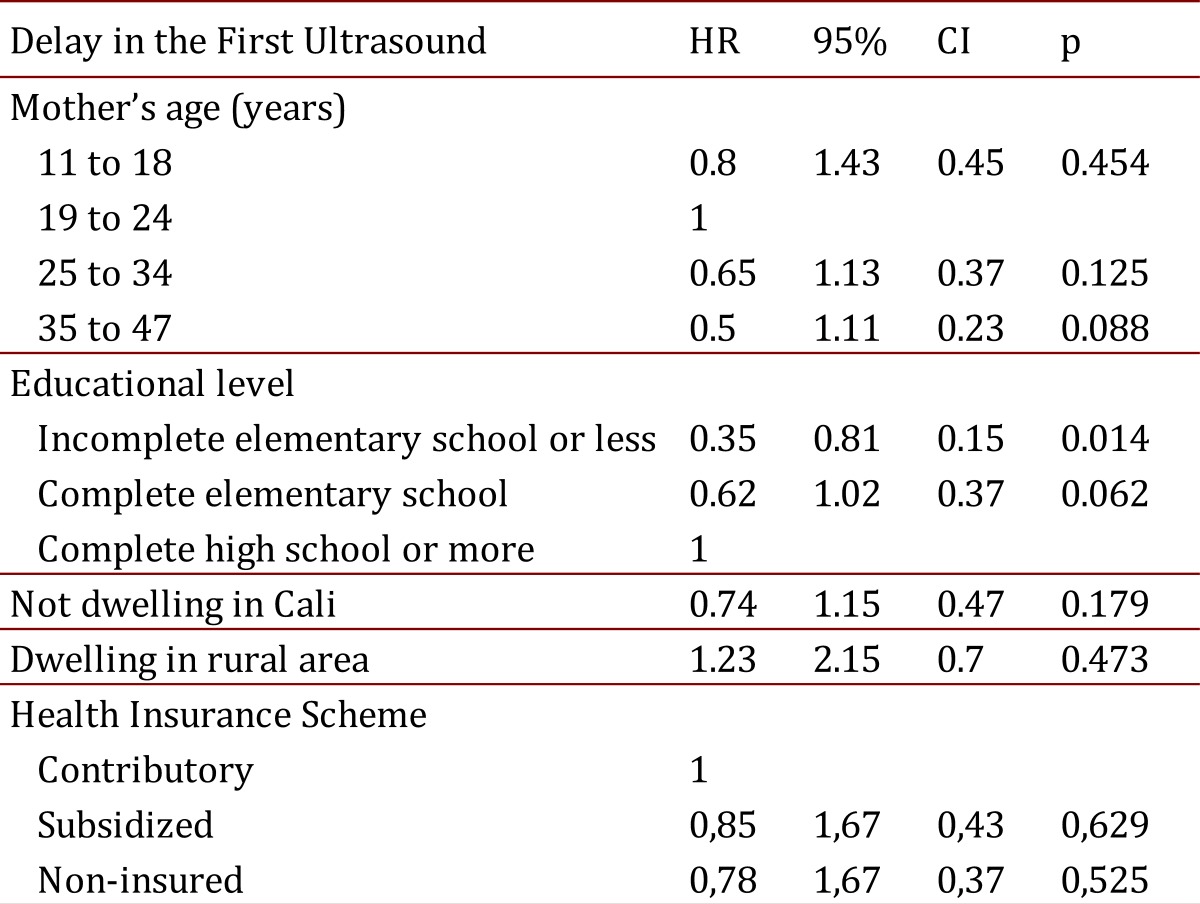
Determinants of the Delay to Access to the First Prenatal Ultrasound during the Pregnancy Control

The most frequently observed congenital defects in the newborns, hospitalized in NICU, were the congenital heart diseases (19.2%); then, the kidney and urinary tract anomalies (17.8%); and thirdly, the *central nervous system* (CNS) defects (14.2%). The congenital defects most frequently detected by the PNUS screening were the CNS anomalies (83.9%), followed by the congenital heart diseases (54.8% of CDDPU). The least diagnosed defects were those in extremities (26.3%) and those in the gastrointestinal tract (18.8%) (See Table 1).

## Discussion

Prenatal diagnosis has evolved with the advancement of ultrasound. Since 1958 when Ian Donald made the first contributions to ultrasound in humans, echography has progressed from 2 and 3 dimensions to multiplanar ultrasound and ultrasound with orthogonal planes, and now to pulsed Doppler, achieving a noninvasive approach to minute anatomical details of embryonic and fetal tissues 3 ^,^ 6, which nowadays allows the detection of most congenital anatomical defects before birth 1 ^,^ 3.

At the time of this study in Colombia, Resolution 412 of 2000 enacted by the Ministry of Health resulted in the publication of guidelines for the process of prenatal diagnoses of congenital defects. It regulated the provision of obstetric ultrasound screening between the 19^th^ and 24^th^ weeks of pregnancy for all pregnant women, and asked for an ultrasound with anatomical detail, or "Level III", for pregnant women with risk factors, or for those that had a basic ultrasound finding that suggested a congenital defect 7.

However, despite the mandatory standard for all social security insurance schemes in Colombia, this study found that one in four mothers of newborns with CDDPU, who were hospitalized in NICU, had no ultrasound performed during the pregnancy. It indicates that Colombia is far from achieving full coverage for ultrasound screening of pregnant women. This study reported that 42.8% of patients had no prenatal diagnosis of their CDDPU, and among them 59.5% had not any PNUS. Thus, lack of PNUS during the pregnancy is a determinant factor for the lack of the prenatal diagnosis of CDDPU, and also, lack of PNUS is a criterion for a bad quality pregnancy control.

The lack of ultrasound during the pregnancy control, in this study, was associated with the maternal age (age groups between 25 and 34 years, and between 35 and 47 years old); and also it was associated with mothers without complete elementary education, which was the strongest association factor (OR: 8.70). These results allow us to infer that women with high risk of having offspring with congenital defects, such as older women or mothers with low educational level, would likely not have access to or adhere to the pregnancy control or to the obstetric ultrasounds. Therefore, they would have less probabilities of having a prenatal diagnosis of congenital anomalies for their fetuses, thus avoiding the appropriate interventions for improving the prognosis of pregnancy and the affected newborns 8 ^-^ 9.

Among mothers who accessed to PNUS, there were no differences in proportions of diagnosis of CDDPU by their type of health insurance. Also, it was found that among all mothers of 35 years or older, who underwent one or more PNUS during their pregnancy controls, at least one CDDPU was detected for such PNUS. This fact suggests that when an ultrasound physician performs a PNUS on a patient with a strong risk of offspring with congenital defects, such as mother's age, it was more likely that the physician would make extended efforts for finding any congenital defect in the ultrasound.

It was also found in the group of women who had one or more PNUS, that the absence of CDDPU diagnosis was associated with having only one PNUS in comparison with having two or more (OR: 4.00). Therefore, having only one PNUS was marginally associated with the subsidized health insurance plan when compared with the contributory insurance (OR: 4.76, *p*= 0.058). A similar trend was found among those that were uninsured, but it was not statistically significant. Additionally, pregnant mothers without health insurance tended to delay twice as long to access to the first PNUS of pregnancy control when compared with mothers belonging to the contributory insurance (HR: 0.51). This trend was also observed for pregnant women belonging to the subsidized insurance, although it was not statistically significant. Additionally, adjusted analyses indicated that these relationships were mainly explained by mother's educational level.

All together, the relationships between lack of prenatal diagnoses of CDDPU with the type of health insurance plan, the number of ultrasound scans and the gestational age at the first ultrasound, indicate that the Colombian state guideline of having only a mandatory obstetric ultrasound between 20 and 24 weeks of gestation was not correct. This situation was effectively changed in subsequent Colombian guidelines on clinical practice for detection and treatment of pregnancy and childbirth complications of 2013, which establish two mandatory ultrasounds during pregnancy, the first between 10 weeks 6 days and 13 weeks 6 days, and the second between 18 weeks and 23 weeks and 6 days. This policy applies to all pregnant women, regardless of their health insurance system 10.

When a congenital defect is detected through ultrasound screening, an echography with anatomical detail should be performed; also, a fetal echocardiogram or a fetal neurosonography may be required. Once the anomaly is confirmed and characterized, a medical protocol should be performed in order to find the cause. For example, performance of a fetal karyotype in chorionic villus, amniotic fluid or umbilical cord blood in cases where it is suspected a chromosomal abnormality or the latter must be ruled out 2 ^,^ 3_._ Also, identify severe cases of congenital defects which are incompatible with life, such as anencephaly or bilateral renal agenesis, and explain the mother's legal right to voluntary abortion, where national legislation permits it 3 ^,^ 11. On the other hand, it is possible to carry out a preventive or therapeutic fetal intervention *in utero* in select cases, such as the release of amniotic bands or laser fetoscopy in fetal transfusion syndrome 1 ^,^ 3. Prenatal diagnoses are necessary for defining the best path for birth according to characteristics and pathologies of the fetus; for example, in patients with myelomeningocele or gastroschisis, where delivery via cesarean section is indicated to prevent complications and improve the prognosis of the patient 12 ^-^ 15. Also, it allows to prepare a multidisciplinary team that will attend to the newborn, in which are specialists such as pediatric surgeons, perinatologists, neonatologists, among others, who will be present, available and prepared for cases that require early medical or surgical management 3 ^,^ 12 ^-^ 16. If there is an accurate prenatal diagnosis, interventions to reduce the neonatal morbidity and mortality can be accomplished. It allows time to prepare the family for having a member with special life conditions, also the newborn would require a prolonged hospital stay and individualized psychomotor stimulation, among other things 1 ^,^ 3 ^,^ 6 ^,^ 13 ^-^ 14 ^,^ 16 ^-^ 17.

In Latin America in general, and in Colombia in particular, in the major cities there are increased numbers of basic and advanced prenatal ultrasound centers which have increased the available coverage for pregnant women from all health insurance schemes, but likely in favor of pregnant women belonging to contributory schemes. However, in this study the coverage of prenatal ultrasound during pregnancy was lower than expected, 25% of mothers had no PNUS; and among the others (i.e. who had at least a PNUS), 23.4% did not have a prenatal diagnosis of CDDPU. Overall, in this study 57.2% of newborns had a prenatal diagnosis of at least CDDPU.

When compared with the results from other studies that also evaluated the prenatal diagnosis of birth defects, an important difference was found from results reported by Gomez et al., who established the presence of a prenatal diagnosis in 32% of newborns with birth defects diagnosed before discharge 5. However, the difference can be explained by the methodology since in this study no cases were reported for those that did not reach the neonatal intensive care unit before death or for those not needing NICU services. In Latin America, Capaña et al. found a prenatal diagnosis in 56% of cases in 18 hospitals in four countries 4 and this result is very similar to the overall results of this study where 57.2% of cases had a prenatal diagnosis of congenital anomalies. Therefore, we believe that results reported herein are similar to others evaluating prenatal diagnoses, which validates our findings.

The sum of the differing factors found here, plus clinician requests for ultrasound testing at gestational ages that are inconsistent with those adequate for establishing prenatal diagnoses, as well as the short time spent on examining each ultrasound, the poor remuneration for each ultrasound performed or for the value of the paid time for the specialist, altogether with norms that are not precise about who can do basic or advanced prenatal ultrasounds, overall are factors reflected on the main results found in this study: an absence of prenatal diagnoses of CDDPU in 42.8.% of patients hospitalized in the intensive care units studied.

## Conclusion

In this study, the proportion of prenatal diagnoses of congenital defects is low; it could be explained by lack of prenatal ultrasound or the absence of an appropriate diagnosis in such ultrasounds. An association was found between the non-performance of prenatal ultrasounds and older mothers with low educational levels. In addition, mothers without health insurance tended to delay twice as long in accessing to the first prenatal ultrasound when compared with other mothers, a fact mainly explained by their low educational level. Adequately funded national policies should be established which ensure access to timely and good quality prenatal ultrasounds for all pregnant women in Colombia, irrespective of their health insurance scheme or other socio-economic factors.
